# Beyond Individual Vulnerability: Recommendations for Structurally Informed Intervention

**DOI:** 10.3390/bs16050751

**Published:** 2026-05-12

**Authors:** Carlos Barros

**Affiliations:** 1 Instituto Politécnico da Lusofonia, 1950-396 Lisboa, Portugal; carlos.barros@ipluso.pt or cbarros@ucp.pt; 2 Research Centre for Communication and Culture, Universidade Católica Portuguesa, 1649-023 Lisboa, Portugal; 3 Aventura Social Team, 1400-415 Lisboa, Portugal

**Keywords:** vulnerability, intersectionality, relational practice, mediation, empowerment, systemic intervention

## Abstract

This research examines expert advice on interventions for populations facing social vulnerability. Based on semi-structured interviews with professionals in psychosocial support, health, education, human geography and public policy, the study employs reflexive thematic analysis to detect common themes in how vulnerability is perceived and managed in practice. The results identify three interconnected clusters: first, viewing vulnerability as a product of structural factors, highlighting issues like institutional fragmentation, bureaucratic obstacles, and policy inconsistencies rather than individual shortcomings; second, emphasising relational and recognition processes, such as trust, active listening, and respect for personal journeys as key to meaningful engagement; and third, focusing on mediation and empowerment tactics, including institutional mediation, critical education, and digital literacy, to improve access and agency without shifting responsibility to individuals. Overall, the findings suggest that effective intervention demands integrated strategies that address structural conditions, relational factors, and empowerment methods. By consolidating expert insights, the study offers empirically based guidance for practice and service organisation, emphasising the need for structurally aware, relationally grounded, and context-sensitive responses to current vulnerabilities.

## 1. Introduction

The growing complexity of social inequalities underscores the inadequacy of individualistic frameworks for understanding social vulnerability. In contexts characterised by job insecurity, increased migration, fragmented public policies, and pervasive technological mediation, vulnerability is better understood as a structurally produced phenomenon. It stems from asymmetrical power relations and institutional mechanisms that unevenly allocate risks, resources, and opportunities.

Recent literature has consistently demonstrated that contemporary forms of inequality are shaped by the interaction between economic restructuring, welfare state transformations, and global mobility, producing new configurations of risk and exclusion (e.g., Standing, 2011; Quesada et al., 2011). In this context, vulnerability cannot be reduced to individual characteristics but must be analysed as the outcome of structural arrangements that differentially position individuals and groups in relation to access to resources, rights, and recognition.

### 1.1. Vulnerability and Intersectionality

The concept of structural vulnerability has been extensively employed to characterise these dynamics, referring to social, economic, cultural, and political conditions that expose certain groups to systematic patterns of exclusion, suffering, and limited agency, regardless of their individual characteristics (Quesada et al., 2011; Farmer, 2004). From this standpoint, vulnerability is not a consequence of personal deficiencies but results from the manner in which social structures regulate access to rights, recognition, and protection. By conceptualising vulnerability as institutionally produced, an implicit appeal for systemic social redress is invoked. This encompasses policies aimed at redistributive justice to guarantee equitable access to resources, as well as recognition-based interventions that acknowledge and address the specific experiences of marginalised populations. Such measures underscore the pressing need for public policies that transcend superficial symptom management and instead target the dismantling of structural mechanisms that perpetuate inequality.

This perspective is strongly developed in the fields of public health, where vulnerability has been analysed in relation to health inequities, access to care, and the social determinants of health. Research in these areas highlights how structural factors—such as poverty, migration status, and institutional discrimination—systematically shape exposure to risk and access to protection, often resulting in avoidable disparities in health outcomes (e.g., Farmer, 2004; Quesada et al., 2011).

Importantly, these approaches are closely linked to Human Rights frameworks, which emphasise the obligation of institutions and states to ensure equitable access to healthcare and social protection (World Health Organization, 2008). From this standpoint, addressing vulnerability is not only a matter of effective intervention but also of justice and rights, requiring structural and policy-level responses.

Intersectionality provides a theoretical framework for understanding how multiple dimensions of inequality overlap and interact. Originally articulated by Crenshaw (1991) in the context of Black women’s experiences in the United States, intersectionality posits that social categories such as race, gender, socioeconomic class, and sexual orientation intersect at the micro level of individual experience. These intersections reflect interlocking systems of privilege and oppression operating at the macro-structural level that have a direct impact on the micro level. Importantly, intersectionality is not a simple additive model of disadvantage; rather, it emphasises that the convergence of multiple social positions produces qualitatively distinct forms of vulnerability. This distinction is particularly relevant in differentiating intersectionality from generic multifactorial or interaction-based explanations. While multifactorial approaches typically describe the accumulation or statistical interaction of independent variables, intersectionality emphasises how social categories are co-constituted within systems of power, producing qualitatively distinct forms of experience that cannot be reduced to additive or moderating effects (Crenshaw, 1989, 1991).

In this sense, intersectionality provides an analytical lens that is not only descriptive but also critical, as it foregrounds how institutional arrangements and power relations shape the conditions under which vulnerability is produced and experienced. Subsequent developments by some authors (e.g., Collins & Bilge, 2020; Garcia & Zajicek, 2022; McCall, 2005) have expanded this framework, demonstrating its relevance for analysing diverse social realities, including migrants, older adults, and LGBTQ+ populations.

Empirical research has illustrated how intersectionality operates through the convergence of multiple axes of inequality in concrete situations. For example, studies in public health and social policy have shown that migrants in precarious labour conditions often face compounded vulnerabilities resulting from the intersection of gender, legal status, and socioeconomic position, which jointly shape their access to healthcare, employment, and social protection (Quesada et al., 2011; Bowleg, 2012). Similarly, research on ageing and digital exclusion demonstrates how older adults with lower educational levels and limited economic resources experience layered forms of disadvantage, particularly in highly technologized institutional environments (van Deursen & van Dijk, 2014; Ge et al., 2025). These examples illustrate that vulnerabilities do not simply accumulate but interact in ways that produce qualitatively distinct experiences of exclusion.

Bowleg (2012) argues that a commitment to social justice renders intersectionality an indispensable analytic lens, as it captures how different identity markers combine to shape lived experiences of marginalisation and resilience. Literature has further demonstrated the value of intersectionality for analysing public policies and institutional practices, showing that ostensibly neutral systems may generate exclusionary effects when they fail to account for intersecting social positions, since they do not fully recognise the effect of the context in individual trajectories (Hankivsky & Cormier, 2011; La Barbera et al., 2023).

Together, structural vulnerability and intersectionality underscore the importance of examining vulnerability at the intersection of multiple systems of power, integrating both micro-level experiences and macro-level institutional arrangements. This perspective aligns closely with ecological models of human development (Bronfenbrenner, 2005), which conceptualise human functioning as the product of dynamic interactions between individuals and their social, institutional, and cultural environments, always considering the temporal dimension. In other words, each individual must be considered in a systemic approach with a context, time, and multi-level interaction that has an impact on their development throughout their life.

At the same time, it is important to avoid essentialising vulnerability as a fixed or inherent condition. As previously noted in the literature, vulnerability is neither binary, nor unitary, nor stable, but varies according to context, time, and the specific forms of harm at stake (Gorovitz, 1994). In this sense, individuals and groups are not inherently vulnerable; rather, they are made vulnerable, to different degrees and in different ways, through particular constellations of structural conditions, institutional arrangements, and social relations.

This implies that vulnerability must always be understood as situated: vulnerability of whom, to what, when, and where. Such an approach allows for a more precise and context-sensitive analysis, avoiding generalisations that may obscure the diversity of lived experiences and the specific mechanisms through which vulnerability is produced and managed in practice.

### 1.2. Empowerment, Literacy, and Mediation as Inclusion Strategies

The promotion of empowerment—understood as the process by which individuals and communities gain control over decisions and actions that affect their lives—constitutes a central pillar of community and psychosocial intervention (Perkins & Zimmerman, 1995). Empowerment has been conceptualised as a multilevel construct operating at the individual, organisational, and community levels, encompassing the development of competencies, active participation, and the transformation of power relations (Rappaport, 1981; Perkins & Zimmerman, 1995; Zimmerman, 2000). From this standpoint, empowerment cannot be reduced to individual psychological strengthening; it necessarily involves structural conditions that enable agency and participation.

Emancipatory education, as formulated by Freire (1972, 1992), is inseparable from processes of empowerment. By fostering critical consciousness, education enables individuals to understand the social conditions shaping their lives and to develop the capacity to intervene in their realities. This dialogical, horizontal approach emphasises listening, recognition, and the co-construction of knowledge—principles that resonate strongly with both critical pedagogy and community psychology.

In contemporary societies, critical literacy—including media and digital literacy—has become increasingly important for inclusion and empowerment. Within this framework, digital literacy should not be understood as an isolated competence, but as a contemporary dimension of structural and institutional participation. In highly technologized environments, access to information, institutional communication, and digital platforms is a crucial factor for social participation. While initial studies focused on participatory skills in digital environments (Jenkins, 2006), recent research shows that digital exclusion is not just about access but also involves unequal use patterns, skills, and support (Ganito, 2018; van Deursen & van Dijk, 2014). Latest reviews indicate that digital exclusion more heavily impacts older adults, people with mental health issues, and socioeconomically marginalised groups, thus deepening existing inequalities (Barros & Ganito, 2024; Ge et al., 2025; Rafi et al., 2019).

In this context, technological transformations add another layer of complexity to vulnerability and power relations, which we cannot identify as a new factor, since at the beginning of the century, Castells (2002) described the emergence of the network society as a reconfiguration of social structures through communication technologies, offering new possibilities for participation while simultaneously amplifying existing inequalities. Without adequate policies for digital inclusion and literacy development, technological expansion risks deepening structural vulnerability by creating new forms of informational exclusion.

In this context, mediation is a critical relational mechanism for translating institutional logics, facilitating access, and building trust. Research on intercultural and institutional mediation demonstrates its effectiveness in reducing power asymmetries and supporting navigation of complex systems, particularly in health and social service contexts (Mejsner et al., 2024; Díaz-Millón & Olvera-Lobo, 2025). These findings corroborate earlier theoretical perspectives that conceptualise mediation as both a relational and political practice, situated at the boundaries between social worlds.

### 1.3. Relational Dimensions and Social Support

The centrality of human relationships runs throughout the literature on vulnerability and intervention. Although originating from distinct research traditions, concepts such as social support, social capital, and social networks share a common relational foundation.

Beyond their descriptive convergence, relational constructs point to a common analytical mechanism: the mediation of access to resources, recognition, and participation through social interaction. In this sense, relational processes are not merely supportive but constitutive of intervention outcomes, shaping both engagement and the effectiveness of institutional responses. This perspective aligns with recognition-based approaches (Honneth, 1996), which emphasise that social integration depends on intersubjective processes of validation and respect. It also reinforces recent findings in psychosocial intervention research, where relational continuity and trust are identified as central mechanisms linking structural conditions to individual trajectories (Heino et al., 2025; Cleece et al., 2025).

Research in developmental and health psychology consistently shows that social support functions as a powerful protective factor in the face of adversity (Cobb, 1976; Cohen & Wills, 1985). Contemporary conceptualisations of resilience emphasise its fundamentally relational nature, describing positive adaptation as emerging through access to supportive relationships that provide meaning, guidance, and resources (Walsh, 2020; Ungar, 2008, 2013).

These relational processes are deeply rooted in psychology. Rogers (1957) emphasised empathy and unconditional positive regard as essential conditions for personal growth, underscoring the transformative power of genuine listening. Recent empirical research in social and psychosocial services indicates that trust, relational continuity, and recognition are not merely supplementary but fundamental to effective intervention (Heino et al., 2025; Cleece et al., 2025), highlighting a critical dimension of human needs.

At the community level, strengthening social networks and social capital remains a key strategy for mitigating structural vulnerability, as communities characterised by trust, reciprocity, and civic engagement provide informal protective networks that buffer against exclusion and marginalisation (Barros & Hanenberg, 2024; Putnam, 1995). In summary, the theoretical landscape indicates that interventions with vulnerable populations should integrate a structural and intersectional understanding of vulnerability with strategies for empowerment, critical education, and cultural or technological mediation.

Nevertheless, there is a lack of empirical studies that systematically examine expert recommendations in these contexts, particularly in ways that articulate theoretical frameworks with concrete intervention practices. Previous research has examined intervention practices in contexts of social vulnerability, particularly highlighting the importance of relational work, institutional mediation, and empowerment strategies in facilitating access to services and promoting inclusion (e.g., Castel, 2017; Meyer, 2014). Studies in community and social psychology further demonstrate that effective interventions depend on the articulation between structural conditions and relational processes, rather than on isolated individual-level approaches (Collins & Bilge, 2020; Meyer, 2014). However, these contributions often remain fragmented across different domains of research, with limited integration between theoretical frameworks and concrete professional practices. This fragmentation points to the need for empirical studies that systematise expert knowledge in a more integrated and operational manner.

This study addresses this gap by identifying and systematising recommendations from experienced professionals for effective interventions with socially vulnerable groups. By focusing on how vulnerability is interpreted and managed in practice, the study seeks to bridge theoretical perspectives with actionable insights for intervention and policy.

## 2. Materials and Methods

This study adopted an exploratory qualitative design based on semi-structured interviews with experts to identify and systematise recommendations for interventions with vulnerable populations. The qualitative method is suitable for in-depth exploration of professional concepts, practices, and guidelines (Charmaz, 2009; Silverman & Patterson, 2021), as well as for capturing the complexity and contextual aspects of vulnerability.

The analytical framework of the study was based on reflexive thematic analysis (Braun & Clarke, 2006, 2019), informed by a post-positivist epistemological orientation. In this context, post-positivism is understood as recognising the existence of an external reality while acknowledging that knowledge about it is necessarily partial and mediated by the researcher’s interpretative framework (Charmaz, 2009; Phillips & Burbules, 2000).

This perspective supports the use of qualitative methods aimed at generating analytically grounded interpretations, emphasising systematic procedures, transparency, and critical reflexivity rather than generalisation.

### 2.1. Participants

This study is part of a larger research-intervention project and draws on expert interviews conducted in two phases. In the first phase in 2022, eight experts were selected for their professional and academic experience in areas related to social vulnerability, psychological and social interventions, medicine, education, human geography, and public policy to help construct research instruments for interventions with transnational families. Participants combined academic activity with professional practice or direct intervention with vulnerable populations.

After this phase, in a second data collection in 2024, five additional experts were interviewed. This extension of the sample was analytically motivated by the emergence of additional topics during the initial analysis. The inclusion of these participants aimed to deepen and consolidate the analysis of these dimensions.

The final sample, therefore, comprised (13) thirteen experts. To preserve confidentiality, participants are identified in the results section using alphanumeric codes (E1–E13).

The participants were between 30 and 60 years old. (*M* = 45; *SD* = 8.5). The interviews lasted between 50 and 95 min, either in person or via video call, as indicated by the participants.

The study was conducted in the Portuguese context, characterised by a welfare system combining universalistic principles with significant institutional fragmentation. This context is particularly relevant for understanding the challenges identified by the participants, especially regarding access to services, bureaucratic complexity, and regional disparities.

Professionals from different regions of Portugal were included in order to capture geographical and institutional diversity. Table 1 presents the range of professional fields represented in the sample.

### 2.2. Procedure

Participants were recruited through a two-step process. In an initial stage, an open online call for participation was disseminated through professional and academic networks, inviting experts with experience in psychosocial intervention, social vulnerability, and related fields to register their interest. Eligibility criteria included professional and/or academic experience in intervention contexts and direct engagement with populations experiencing social vulnerability.

Following this initial recruitment, snowball sampling was employed, whereby participants were invited to suggest additional experts whose profiles and experience were relevant to the aims of the study. This strategy was used to broaden the range of expertise represented and to access information-rich cases that might not be reachable through open recruitment alone.

Data were collected through interviews focusing on participants’ conceptualisations of vulnerability, challenges in intervention, professional experiences, and recommendations for working with socially at-risk populations. Particular attention was paid to the main social issues participants encountered in their professional contexts.

All interviews were documented as detailed analytical notes and subsequently organised and systematised for analysis. The use of analytical notes rather than full verbatim transcription reflects the study’s applied and exploratory nature, as well as its focus on identifying key dimensions and recommendations relevant to research and intervention. These analytical notes were expanded immediately after each interview to preserve contextual detail and reflexive observations. To enhance the credibility of the data, key points were summarised and validated with participants at the end of each interview (member checking technique), allowing for clarification or correction where necessary (Lincoln & Guba, 1985).

### 2.3. Data Analysis

Data analysis followed the phases proposed by Braun and Clarke (2006, 2019), including familiarisation with the data, initial coding, and the construction and definition of themes. Coding was conducted using NVivo (Version 14).

To enhance the rigour and validity of the qualitative analysis, the study followed established principles of qualitative research, including transparency in analytical procedures, iterative engagement with the data, and reflexive consideration of the researcher’s role (Charmaz, 2006, 2009; Braun & Clarke, 2019; Small & Calarco, 2022). Consequently, the analysis adhered to the criteria of qualitative trustworthiness—specifically credibility, dependability, and confirmability (Lincoln & Guba, 1985). To operationalize these criteria, coding decisions were documented throughout the process and an audit trail was maintained, ensuring transparency and consistency in the development of themes (Nowell et al., 2017).

The analysis was conducted through an iterative and reflexive process. Initial codes were generated inductively from the data and progressively refined through constant comparison across interviews (Charmaz, 2006). Themes were not imposed a priori but developed through sustained engagement with recurring patterns, while being interpreted in dialogue with the theoretical framework and, simultaneously, with the research project’s supervisor’s team to avoid bias.

To enhance analytical credibility, coding decisions were revisited across multiple stages, and alternative interpretations were critically examined. NVivo software (14 edition) supported data organisation and traceability of coding processes, contributing to transparency in the analytical procedure. At a later stage of analysis, themes were organised into interpretative clusters. The construction of these clusters was supported by NVivo’s cluster analysis tools, drawing on coding similarity between nodes and visual representations of thematic proximity. This process enabled the grouping of themes into clusters reflecting different levels and logics of intervention.

Overall, these strategies align with contemporary standards for qualitative rigour, privileging depth of interpretation, coherence, and methodological transparency (Small & Calarco, 2022). Throughout the analytical process, reflexive awareness of the researcher’s interpretative role was maintained, recognising that themes are constructed through engagement with the data rather than discovered as objective entities (Braun & Clarke, 2019).

## 3. Results and Discussion

The thematic analysis of the interviews resulted in the identification of three interrelated interpretative clusters: (1) Structural and systemic conditions, (2) Relational and recognition processes, and (3) Mediation and empowerment strategies. These clusters synthesise complementary dimensions of intervention and reflect a shared understanding among participants of vulnerability as a phenomenon that is simultaneously structural, relational, and intersectional.

Each cluster corresponds to a distinct, though interconnected, level of intervention and allows the empirical material to be articulated with the relevant literature on vulnerability, intersectionality, recognition, and empowerment. Table 2 presents a summary of the clusters, their analytical focus, and the main themes identified.

### 3.1. Cluster 1—Structural and Systemic Conditions: Vulnerability as a Social Product

In all the interviews, participants consistently rejected individualising interpretations of vulnerability, instead framing it as the outcome of structural inequalities, institutional failures, and fragmented public policies. This next excerpt illustrates this:

“There is always a tendency to ask what the person did or failed to do, when the problem is in the system that does not respond. We [as doctors] need to be aware of a more systematic approach”.

(E3)

The structural understanding of vulnerability aligns with views that see risk exposure as rooted in social, political, and institutional systems rather than individual behaviour (Quesada et al., 2011; Farmer, 2004). Participants clearly shifted blame away from individuals, instead highlighting institutional factors, echoing criticisms of moralising perspectives on social exclusion.

About two-thirds of participants emphasised that formal access to rights does not guarantee their effective realisation, particularly for individuals experiencing prolonged precarity, migration, or cumulative exclusion. We illustrate this point with a phrase to a psychosocial intervention expert:

“The rights exist, but between existing and actually being able to access them, there is a huge distance, full of bureaucracy and invisible barriers. To have minimal guidance in the normative ways of state mechanisms, we need to fill a lot of documents that state the emergency”.

(E7)

Another participant highlighted how vulnerability is shaped by cumulative institutional constraints:

“Sometimes it’s not one barrier, it’s many small ones that accumulate—documents, deadlines, requirements—until people simply give up”.

(E10)

This account aligns with intersectional analyses that show how multiple dimensions—such as legal status, socioeconomic position, and institutional literacy—intersect to shape interactions with public systems (Crenshaw, 1991; Bowleg, 2012).

Participants’ descriptions concretely illustrate how institutional fragmentation and administrative practices contribute to what recent literature defines as structural vulnerability, operationalized through everyday encounters with systems of welfare, health, and social protection (La Barbera et al., 2023; Metzl et al., 2018).

Importantly, nearly half of the experts highlighted that institutional responses are often discontinuous and poorly coordinated, producing cumulative effects that intensify vulnerability rather than mitigate it. These findings underscore the need for interventions that address systemic determinants and institutional logics, rather than focusing exclusively on individual adaptation.

### 3.2. Cluster 2—Relational and Recognition Processes: The Centrality of the Relationship

The second cluster emphasises the central role of relational processes in intervention with vulnerable populations. All participants consistently described trust, active listening, and respect for individual rhythms as indispensable for effective engagement and change. The next statement reflects a shared understanding among participants that relational work is not an auxiliary component of intervention, but its very foundation: “Without a relationship there is no intervention. If there is no trust, nothing else works” (E2).

Several experts noted that many individuals in situations of vulnerability carry histories of institutional interactions characterised by judgement, distrust, or dehumanisation, which make these individuals opt to close in on their problems and not be open to possible solutions, as we can see in this excerpt of E11:

“Many people have been ignored or treated like numbers. When they feel this again, they shut down completely because they say [in therapy] that they feel minimised and almost like outsiders.”

Such accounts align with theoretical perspectives on recognition, which emphasise that experiences of misrecognition undermine agency and participation (Mejsner et al., 2024; Díaz-Millón & Olvera-Lobo, 2025). From this standpoint, the relationship between professional and service user functions as a space for symbolic recognition that can counteract prior experiences of invisibility.

Also, almost half of the experts explicitly rejected paternalistic or prescriptive approaches, emphasising the importance of respecting individuals’ timing and autonomy, as we can see in E1 narrative:

“You cannot impose change. You have to walk at the person’s pace, otherwise you just reproduce the same violence patterns”.

Similarly, another professional emphasised the importance of recognition in rebuilding trust: “When people feel that someone is truly listening to them without judgment, that’s when they begin to engage again” (E6).

These findings align with the literature showing that trust and relational continuity are critical to engagement and effectiveness in social and psychosocial services (Heino et al., 2025; Cleece et al., 2025). The data suggest that relational processes mediate access to resources and support, reinforcing the view that recognition is a core mechanism of social justice rather than merely an ethical add-on.

### 3.3. Cluster 3—Mediation and Empowerment Strategies: Education, Literacy, and Agency

The third cluster brings together recommendations on mediation, education, and empowerment, highlighting these dimensions as transversal strategies for inclusion. Participants described a substantial part of their work as helping individuals navigate complex institutional systems.

It is important to note that a significant proportion of respondents emphasised the importance of mediation, either directly or by describing its role in their professions. We include an example from the narrative of E9, which indicates:

“Very often we act as translators of the system: we explain, accompany, and help overcome barriers to find a path to well-being”.

This description corresponds closely to contemporary research on institutional and intercultural mediation, which conceptualises mediators as agents who reduce power asymmetries and facilitate access to rights by translating bureaucratic and symbolic codes (Mejsner et al., 2024; Díaz-Millón & Olvera-Lobo, 2025). Participants emphasised that empowerment should not be understood as the mere transmission of information, but as the development of critical understanding and autonomy.

This perspective aligns with empowerment theory as a multilevel process that integrates individual agency with structural conditions (Zimmerman, 2000; Perkins & Zimmerman, 1995). Participants were careful to stress that empowerment strategies must not shift responsibility onto individuals in the absence of institutional support.

Education and literacy—particularly institutional and digital literacy—were repeatedly identified as key resources for promoting inclusion. Seeing the observation of E5: “If people don’t understand the language of institutions, they are excluded before they even start”, we can connect with recent research demonstrating how digital and institutional exclusion reproduce broader social inequalities, particularly among already marginalised groups (van Deursen & van Dijk, 2014; Ge et al., 2025).

Almost all participants emphasised that literacy initiatives must be accompanied by mediation and sustained support, rather than framed as individual competencies detached from context.

### 3.4. Integrative Interpretation of Clusters

Taken together, the three interpretative clusters highlight that vulnerability emerges from the interaction between structural conditions, relational processes, and mediation practices. While analytically distinct, these dimensions are empirically intertwined in professional practice.

Rather than operating as separate levels, structural constraints shape relational encounters, while relational processes condition access to mediation and empowerment strategies. In turn, mediation practices constitute concrete mechanisms through which structural barriers are navigated and, in some cases, partially transformed. This integrated perspective reinforces the need for interventions that are simultaneously structurally informed, relationally grounded, and operationally oriented.

Cluster 1 situates vulnerability in structural and institutional arrangements, rather than in individual deficits. Participants’ emphasis on bureaucratic barriers, fragmented services, and policy discontinuity aligns with conceptualisations of structural vulnerability that locate patterned exposure to harm within political and institutional systems (Farmer, 2004; Quesada et al., 2011). Recent empirical work further demonstrates how such vulnerability becomes visible in everyday institutional encounters, including administrative gatekeeping and unequal access to protective resources (Metzl et al., 2018).

Cluster 2 highlights relational processes as central mediators of engagement with institutions. Participants’ focus on trust, active listening, and respect for individual pace is consistent with recognition-based perspectives, which emphasise that misrecognition undermines agency and participation (Honneth, 1996). Studies in psychosocial and social services similarly show that trust and relational continuity are key conditions for effective intervention, particularly in contexts of prior institutional mistrust (Heino et al., 2025; Cleece et al., 2025).

Cluster 3 emphasises mediation and empowerment as practices through which structural constraints are navigated concretely. Participants’ framing of empowerment as developing critical understanding—rather than transferring responsibility—aligns with empowerment theory as a multilevel process linking individual agency to organisational contexts (Perkins & Zimmerman, 1995; Zimmerman, 2000). Their accounts of translation and accompaniment resonate with recent research on institutional mediation, which shows its role in reducing power asymmetries and facilitating access to services (e.g., Díaz-Millón & Olvera-Lobo, 2025).

Across clusters, institutional and digital literacy emerge as transversal resources that can either enable participation or reproduce exclusion, depending on the availability of support. This observation is consistent with evidence that digital exclusion increasingly reflects inequalities in skills and support rather than access alone (van Deursen & van Dijk, 2014; Ge et al., 2025). Overall, the clusters suggest that vulnerability is structurally produced, relationally mediated, and practically negotiated through mediation and empowerment strategies. Beyond their applied relevance, these findings contribute to theoretical discussions on vulnerability by empirically illustrating how structural, relational, and mediational dimensions are co-constitutive in practice. This integrative perspective supports a more operational understanding of vulnerability as a dynamic and context-dependent process.

### 3.5. Recommendations for Professional Practice

These recommendations should be interpreted in light of the specific institutional and policy context in which the study was conducted, while also offering transferable insights for similar welfare and intervention systems.

Firstly, the results suggest that professionals working with vulnerable populations should adopt a structural and intersectional reading of the situations they deal with. This implies recognising that individual difficulties often reflect systemic barriers, such as complex bureaucracies, restrictive eligibility criteria, or disjointed institutional responses.

Secondly, the centrality that specialists attribute to relational processes points to the need for practices grounded in active listening, trust-building, and recognition of subjects’ lived experiences. The professional-user relationship emerges as a fundamental space for symbolic mediation, capable of counteracting trajectories of institutional delegitimisation. Thus, it is recommended that the initial and continuing training of professionals integrate relational, reflective, and ethical skills.

A third recommendation concerns the role of mediation and training as cross-cutting axes of intervention. The results indicate that supporting the navigation of institutional systems, promoting institutional and digital literacy, and developing critical skills are essential practices for expanding the autonomy and agency of individuals. It is important to emphasise that these strategies should be understood as situated and monitored processes, rather than transfers of individual responsibility.

### 3.6. Recommendations for Public Policy

At the public policy level, the results reinforce the need for integrated, intersectoral approaches capable of addressing the complexity of contemporary vulnerabilities.

It is recommended that policies incorporate a cross-cutting perspective, from diagnosis through implementation to evaluation, recognising how different axes of inequality intersect to produce vulnerability. This implies reviewing administrative criteria, access mechanisms and institutional practices which, although apparently neutral, produce cumulative discriminatory effects.

The results also point to the importance of investing in education, literacy and mediation policies, including digital literacy, as structural components of social inclusion. These dimensions should be integrated in a coordinated manner with social protection, health, housing and employment policies, avoiding one-off or short-term solutions.

Finally, the data suggest that effective policies should recognise and value relational and mediation work as central, rather than ancillary, functions of social intervention.

### 3.7. Limitations and Suggestions for Future Studies

This study has some limitations that should be considered when interpreting the results. The qualitative and exploratory nature of the research, based on a limited number of interviews with experts, does not allow for generalisations. However, this methodological choice enabled an in-depth analysis of professional recommendations and practices in contexts of vulnerability.

Secondly, while focusing on expert perspectives is valuable for understanding intervention guidelines and principles, this approach does not directly incorporate the voices of individuals experiencing vulnerability. Future studies could benefit from participatory methodologies that integrate these individuals’ lived experiences. Adopting co-research approaches with vulnerable populations would align with the empowerment ethic advocated in this study, enabling those affected by social vulnerabilities to actively participate in the research process and ensuring their perspectives are represented.

Additionally, the use of analytical notes rather than full verbatim transcription may limit the depth of interpretative analysis. While this approach aligned with the study’s applied focus, future research could benefit from more detailed transcription and triangulation strategies to further enhance analytical robustness.

While intersectionality offers a valuable framework for understanding overlapping forms of disadvantage, it has also been subject to criticism, particularly regarding potential interpretative subjectivity and limited methodological clarity in some qualitative applications. Some authors also note challenges in empirically operationalising intersecting dimensions. In this study, intersectionality is used as an analytical lens to support interpretation rather than as a strictly operationalised framework, and findings should therefore be considered within this interpretative scope.

Future research could delve deeper into specific contexts or use longitudinal methodologies to analyse vulnerability dynamics over time.

Finally, future studies could more systematically explore the impact of the mediation, empowerment, and critical education strategies identified in this study, contributing to the development of more integrated and sustainable intervention models.

## 4. Conclusions

This study aimed to systematise expert recommendations for intervention with populations experiencing social vulnerability, drawing on qualitative interviews analysed through reflexive thematic analysis. The findings identified three interrelated clusters of recommendations addressing structural and institutional conditions, relational and recognition processes, and mediation and empowerment strategies. Together, these clusters offer a coherent account of how vulnerability is understood and addressed in professional practice.

Experts agree that tackling vulnerability requires more than just individual efforts. They highlight the importance of examining institutional structures, fragmented services, and policy gaps that influence access to rights and resources. However, they also stress that understanding these structures alone is not enough; ongoing relational work grounded in trust, active listening, and respect for people’s experiences is essential. Practices like mediation and empowerment are practical strategies to enhance access and agency, especially when institutional and digital literacy levels vary.

## Figures and Tables

**Table 1:** Composition of the Sample by Professional Field.

	*N*
Age (M; SD)	45; 8.5
Professional Field	
Psychology	4
Social Work	2
Medicine	2
Education	1
Public Policy	1
Human Geography	2
Sociology	1
Total	13

**Table 2:** Interpretative clusters.

Cluster	Analytical Focus	Core Themes Identified with Number of References
Cluster 1	Structural and institutional production of vulnerability	Structural inequalities (16); fragmented policies (9); bureaucratic barriers (12); gap between formal rights and effective access (11); rejection of individualising explanations (12)
Structural and systemic conditions		
Cluster 2	Centrality of relational dynamics in intervention	Trust and active listening (15); respect for individual pace (11); non-paternalistic approaches (7); recognition of subjectivity (10); relationship as a condition for intervention (14)
Relational and recognition processes		
Cluster 3	Mediation, education, and empowerment as transversal mechanisms	Institutional/organisational mediation (11); translation of systems (13); institutional and digital literacy (10); critical education (7); supported autonomy and agency (15)
Mediation and empowerment strategies		

## Data Availability

The datasets generated and analysed in this study are not publicly available because they are part of post-doctoral research with ethical protections for participants’ interviews. However, general data can be obtained from the corresponding author upon reasonable request. Note that the transcribed interviews in this research are in Portuguese.
